# Placental pathological findings and their association with maternal and fetal clinical outcomes: A cross-sectional study

**DOI:** 10.18502/ijrm.v22i10.17662

**Published:** 2024-12-02

**Authors:** Soheila Sarmadi, Elham Mirzaian, Fatemeh Nili, Fatemeh Khalafrezaei

**Affiliations:** ^1^Department of Pathology, Yas Women Hospital, Tehran University of Medical Sciences, Tehran, Iran.; ^2^Department of Pathology, Shariati Hospital, Tehran University of Medical Sciences, Tehran, Iran.; ^3^Department of Pathology, Imam Khomeini Hospital Complex, Tehran University of Medical Sciences, Tehran, Iran.; ^4^Department of Pathology, Kosar Hospital, Semnan University of Medical Sciences, Semnan, Iran.

**Keywords:** Placenta, Histopathology, Pregnancy complications, Maternal health, Fetal outcome.

## Abstract

**Background:**

Placental pathological changes can occur in both normal and high-risk pregnancies, leading to adverse maternal and neonatal outcomes.

**Objective:**

This study aimed to investigate the relationship between placental histopathological findings and maternal and fetal clinical outcomes, as well as to determine if there is an association between maternal comorbidities and placental pathologies.

**Materials and Methods:**

In this study, 250 placenta samples were evaluated. The slides and paraffin blocks were retrieved from the archive of the pathology department of Shariati and Yas hospitals, Tehran, Iran. The placental histopathological findings were analyzed in relation to maternal and fetal clinical data.

**Results:**

The average age of pregnant women was 31.84 yr. The average gestational age at birth was 28 wk. The most prevalent pathological finding was maternal vascular malperfusion, observed in 59.6% of cases. The presence of nucleated red blood cells (NRBC) in umbilical cord blood vessels was observed in 16.4% of cases. Fetal vascular malperfusion was significantly associated with intrauterine fetal demise. Maternal inflammatory response was associated with premature rupture of membranes. Maternal vascular malperfusion and the presence of NRBC in umbilical cord blood vessels was significantly associated with pre-eclampsia and preterm labor. Furthermore, a history of maternal malignancy was associated with placental infarction and the presence of NRBC in umbilical cord blood vessels.

**Conclusion:**

The findings of this study underscore the importance of evaluating placental histopathological findings in relation to maternal and fetal clinical outcomes. Therefore, understanding these placental pathological changes will be crucial in predicting and preventing complications in subsequent pregnancies.

## 1. Introduction

The placenta is a highly specialized organ during pregnancy that facilitates the essential conditions for the growth and development of the fetus. It functions as a maternal-fetal interface responsible for crucial processes such as nutrition, waste removal, and gas exchange between the mother and the developing fetus (1). Unfortunately, placental pathology often goes unnoticed and receives inadequate attention. However, it must be acknowledged that a comprehensive evaluation of pregnancy complications on both the mother and the fetus cannot be accomplished without a thorough examination of the placenta (2). Many adverse pregnancy outcomes are determined during the intrauterine life of the fetus, leading to the notion of the placenta serving as a “diary of intrauterine life" in certain studies (3). Abnormal placental functioning and development significantly increase the risk of various complications in both the mother and the fetus, including pre-eclampsia, fetal growth restriction, pregnancy loss, and stillbirth. These complications can also result in maternal morbidity and mortality (4).

Notably, pathologies affecting the placenta, umbilical cord, or membrane contribute to a considerable proportion (11–65%) of stillbirth cases (5, 6). The reasons for conducting a pathological examination of the placenta encompass identifying the underlying causes of adverse pregnancy outcomes, such as fetal death, identifying cases with a high likelihood of recurrence in subsequent pregnancies, and identifying cases necessitating immediate clinical intervention (7, 8). According to the Amsterdam consensus conference, placental pathologic lesions can be categorized into 4 main groups: acute inflammation, chronic inflammation, maternal vascular malperfusion (MVM), and fetal vascular malperfusion (FVM) (5, 9). Given that the pathological examination of placenta samples can play a pivotal role in managing subsequent pregnancies and preventing adverse outcomes. Owing to the conflicting findings in different studies regarding the correlation between histopathological placental findings and the clinical condition of both the mother and the fetus, we embarked on a 1 yr investigation to explore this relationship more comprehensively.

Therefore, this study aimed to investigate the macroscopic and microscopic findings of the placenta and their relationship with the clinical findings of the mother and fetus.

## 2. Materials and Methods

### Study design

This cross-sectional study evaluated a total of 250 placenta samples obtained from natural delivery or cesarean section between March 2020 and February 2021. The slides and paraffin blocks were retrieved from the archive of the Pathology Department at Shariati and Yas hospitals, Tehran University of Medical Sciences, Tehran, Iran. Samples related to the tumoral and placenta accrete spectrum were excluded from the study.

### Macroscopic evaluation

Macroscopic findings, including placental weight, presence of calcification, infarct, and retroplacental hemorrhage/hematoma, were recorded based on the previous pathology reports.

### Microscopic evaluation

H&E stained slides from each placenta sample, including the umbilical cord, placental membranes, and maternal and fetal surfaces, were reviewed by 2 expert pathologists. Histopathological findings, such as evidence of inflammation, necrosis, stromal fibrosis, syncytial knots, and other relevant findings, were recorded for each sample.

### Clinical data collection

The mother's clinical characteristics, including age, gestational age, pregnancy-related diseases, chronic systemic diseases, infection symptoms, and history of malignancy, were extracted from the clinical documents. Fetal complications, including intrauterine growth restriction (IUGR), premature rupture of membranes (PROM), stillbirth, and multiple gestations, were obtained from the mother's hospitalization records.

### Ethical Considerations

This study was conducted without any intervention on the participants, and no additional costs were imposed. The results were reported in a manner that ensured participant anonymity and confidentiality. Consequently, this study adheres to ethical standards. The Ethics Committee of Tehran University of Medical Sciences, Tehran, Iran has approved this study (Code: IR.TUMS.MEDICINE.REC.1400.693).

### Statistical Analysis

The Statistical Package for the Social Sciences (IBM SPSS Statistic Version 23, IBM Inc., Chicago, IL) was used to analyze the data. The results for qualitative variables were calculated and reported as frequency and percentage, while the results for quantitative variables were presented as mean and standard deviation. Statistical tests, such as independent *t* test, Mann-Whitney, and Chi-square test, were employed to analyze both quantitative and qualitative data. A p-value < 0.05 was considered statistically significant.

## 3. Results

A total of 250 placenta samples were evaluated in this study. The mean age of pregnant women was 31.8 yr, ranging from 15–52 yr. Among the infants, 203 (81.2%) were born preterm, while 47 (18.8%) were born at term. The average gestational age at birth was 28 wk. Table I presents the frequency of clinical findings and maternal complications. Fetal complications included intrauterine fetal demise (IUFD), IUGR, PROM, and twin gestation.

Table II provides the frequency of fetal complications. Histopathological evaluation of the placentas revealed the following findings: MVM including accelerated villus maturation, distal villous hypoplasia, and placental infarction. FVM, placental calcification, nucleated red blood cells (NRBCs) in umbilical cord blood vessels in the second half of pregnancy, maternal inflammatory response (MIR), fetal inflammatory response, and villitis of unknown etiology.

**Figure 1:**
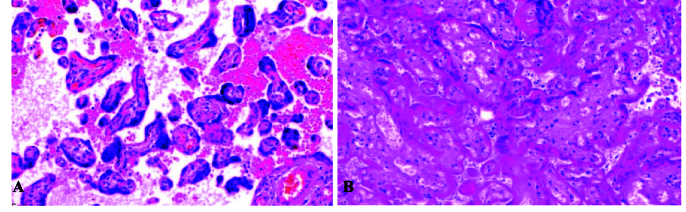
A) Accelerated villus maturation (decreased villous size, increased syncytial knot), and distal villous hypoplasia, B) Placental infarction (H&E, x200).

**Figure 2:**
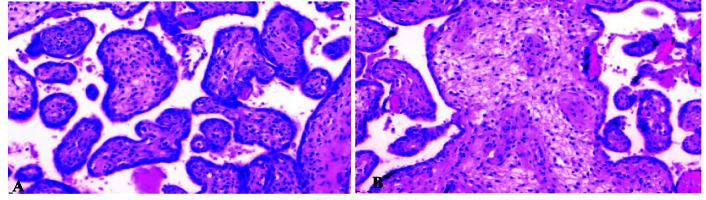
A) Villous stromal-vascular karyorrhexis, B) Stem villous vascular obliteration (H&E, x200).

Additional placental pathologic findings included perivillous fibrin deposition, chorangiosis, placental mesenchymal dysplasia, delayed villous maturation, and intra-villous hemorrhage. Placental hypoplasia, defined as placental weight below the 10 ${\;}^{\text{th}}$ percentile, was observed in 12 cases (4.8%). The frequency of these placental pathologic findings is presented in table III, and examples of placental histopathological findings can be seen in figures 1 and 2.

Table IV summarizes the association between placental pathological findings and the maternal and fetal clinical consequences. No statistically significant relationship was observed between any of the placental pathological findings and maternal age, gestational diabetes, chronic systemic diseases (such as diabetes and hypertension) in the mother, IUGR, or placental weight. Furthermore, no statistically significant relationship was found between placental weight and any of the background characteristics or pregnancy-related complications.

**Table 1:** The frequency of clinical findings and maternal complications

**Variables**		**Frequency**
**Mean age**		31.8
**Gestational age**		
	**Preterm**	203 (81.2)
	**Term**	47 (18.8)
**Pre-eclampsia**		51 (20.4)
**Gestational diabetes**		16 (6.4)
**Diabetes mellitus**		2 (0.8)
**Hypertension**		2 (0.8)
**History of malignancy**		4 (1.6)
**Others**		8 (3.2)
Data presented as n (%)		

**Table 2:** The frequency of fetal complications

**Variables**	**Frequency**
**IUFD**	27 (10.8)
**IUGR**	90 (36)
**PROM**	32 (12.8)
**Twin gestation**	43 (17.2)
Data presented as n (%). IUFD: Intrauterine fetal demise, IUGR: Intrauterine growth restriction, PROM: Premature rupture of membranes	

**Table 3:** The frequency of placental pathologic findings

**Variables**	**Frequency**
**MVM**	149 (59.6)
**AVM**	67 (26.8)
**DVH**	5 (2)
**AVM & DVH**	48 (19.2)
**Placental infarction**	29 (11.6)
**FVM**	6 (2.4)
**Placental calcification**	20 (8)
**NRBCs**	41 (16.4)
**MIR**	19 (7.6)
**FIR**	4 (1.6)
**VUE**	3 (1.2)
**Others**		
	**PFD**	6 (2.4)
	**Chorangiosis**	6 (2.4)
	**PMD**	2 (0.8)
	**DVM**	2 (0.8)
	**IVH**	1 (0.4)
**Placental hypoplasia**		12 (4.8)
Data presented as n (%). MVM: Maternal vascular malperfusion, AVM: Accelerated villus maturation, DVH: Distal villous hypoplasia, FVM: Fetal vascular malperfusion, NRBCs: Nucleated red blood cells, MIR: Maternal inflammatory response, FIR: Fetal inflammatory response, VUE: Villitis of unknown etiology, PFD: Perivillous fibrin deposition, PMD: Placental mesenchymal dysplasia, DVM: Delayed villous maturation, IVH: Intra-villous hemorrhage		

**Table 4:** Association between placental pathological findings and maternal and fetal clinical outcomes

**Association**	**P-value**
**Preterm birth and MVM**	0.002
**Preterm birth and the presence of NRBCs in the umbilical cord blood vessels**	0.040
**Pre-eclampsia and MVM**	0.001
**Pre-eclampsia and the presence of NRBCs in the umbilical cord blood vessels**	0.001
**History of malignancy in the mother and placental infarction **	0.016
**History of malignancy in the mother and the presence of NRBCs in the umbilical cord blood vessels**	0.001
**FVM and IUFD**	0.001
**MIR and PROM**	0.001
**Twin gestation and PFD**	0.025
**Twin gestation and chorangiosis**	0.025
**Twin gestation and IVH**	0.025
Chi-square test. MVM: Maternal vascular malperfusion, NRBCs: Nucleated red blood cells, FVM: Fetal vascular malperfusion, MIR: Maternal inflammatory response, IUFD: Intrauterine fetal demise, PROM: Premature rupture of membranes, PFD: Perivillous fibrin deposition, IVH: Intra-villous hemorrhage	

## 4. Discussion

Pathological changes in the placenta have been extensively reported, particularly in pregnancies classified as high-risk or involving maternal and neonatal morbidities. This raises 2 fundamental questions: first, whether these placental pathologies are associated with adverse maternal and fetal outcomes, and second, whether underlying maternal comorbidities or diseases during pregnancy can be linked to the development of placental pathologies.

In this study, we aimed to investigate the relationship between placental pathology and clinical findings in both the maternal and fetal populations. Initially, our analysis focused on determining the frequency of common placental pathologies observed during pregnancy. Notably, the most prevalent pathological change identified was MVM, accounting for 59.6% of cases. This was followed by the presence of NRBCs in umbilical cord blood vessels, which was observed in 16.4% of cases.

It is imperative to note that the diagnosis of MVM cannot be solely based on a single pathological finding within the placenta. Instead, it encompasses a comprehensive evaluation of macroscopic and microscopic placental features. Macroscopically, MVM is characterized by placental hypoplasia, placental infarction, and retroplacental hemorrhage or hematoma. On a microscopic level, villous lesions such as accelerated villous maturation (indicated by decreased villous size and increased syncytial trophoblast knot), distal villous hypoplasia, and decidual vascular lesions are considered significant indicators of MVM. Due to this multifaceted diagnostic approach, determining the exact frequency of MVM based on existing literature becomes challenging (10). In line with previous studies, placental pathologies have been closely related to maternal and fetal morbidities. The present study highlights a significant relationship between FVM and IUFD. Various histological patterns have been identified in FVM, including thrombosis of the fetal chorionic plate or umbilical cord vessels, avascular villi, villous stromal-vascular karyorrhexis, and stem villous obliteration (fibromuscular sclerosis) (11, 12).

Moreover, the study reveals a significant correlation between MIR and PROM. Additionally, MVM and the presence of NRBC in the umbilical cord vessels during the second half of pregnancy are strongly associated with the development of pre-eclampsia. Furthermore, the preterm condition of the newborn is significantly linked to both MVM and the presence of NRBC in the umbilical cord blood vessels during the latter half of gestation.

Several studies have investigated the relationship between various placental pathological changes and maternal and neonatal morbidities. For instance, a study demonstrated that MVM was found to be associated with recurrent preterm birth (13).

In another study, a significant correlation was observed between neonatal sepsis and placental inflammation as well as villitis of unknown etiology. Additionally, twin pregnancies were found to be associated with MVM (14).

One study reported that marked placental infarction was significantly linked to low APGAR scores and perinatal deaths. An increased presence of syncytial knots, fibrinoid degeneration, vasculo-syncytial membrane paucity, and stromal fibrosis were also identified as factors associated with higher perinatal mortality (15).

Furthermore, another study revealed that cases of gestational diabetes mellitus exhibited significantly higher neonatal weight, placental weight, and placental diameter when compared to normal gestation. Conversely, cases of maternal anemia, pregnancy induced hypertension, and IUGR showed decreased neonatal weight, placental weight, and placental diameter (16). In a study, it was observed that mothers with histologic chorioamnionitis delivered at a very lower gestational age (17). Similarly, another study found a strong association between chorioamnionitis and funisitis with various adverse outcomes including PROM, very low birth weight, neonatal sepsis, and cardiac and neurological complications (18). Our study also revealed a significant correlation between the MIR and PROM.

One study demonstrated a higher incidence of chorioamnionitis in preterm neonates, whereas IUGR neonates exhibited a greater prevalence of villitis. Additionally, a high incidence of chorioamnionitis, funisitis, and villitis was observed in stillborn neonates (19). Another study conducted in Iran found that preterm deliveries were more likely to exhibit placental calcification, which was significantly higher compared to term deliveries. Inflammatory lesions were present in 60% of cases with early preterm labor, and both placental calcification and inflammatory lesions were associated with preterm labor (20).

The pathological examination of the placenta serves as an autopsy of pregnancy, providing valuable insights into various aspects of reproductive health. One of the primary reasons for conducting a pathological examination of the placenta is to elucidate the underlying causes of adverse pregnancy outcomes. The information gathered from this examination can be instrumental in managing future pregnancies (21). Furthermore, examining placental samples can help identify previously undetected diseases, conditions with a high likelihood of recurrence in subsequent pregnancies, and specific explanations for adverse outcomes, such as fetal death. However, not all pregnancies with unfavorable outcomes are associated with pathological lesions of the placenta, and the presence of such lesions does not necessarily result in unfavorable outcomes (8).

A study conducted in 2022 reported that placental pathology was present in 26.2% of normal pregnancies and 73.8% of complicated pregnancies (10). Therefore, even in cases of normal and uncomplicated pregnancies, the presence of placental pathologies can be expected in approximately a quarter of cases.

Both the College of American Pathologists and the Royal College of Pathologists recommend the pathological examination of the placenta in all cases of adverse pregnancy outcomes, as well as when there is an underlying maternal disease, or any gross pathological lesions observed during the examination of the placenta (4, 22). This examination involves a comprehensive evaluation of the umbilical cord, membranes, and placental disc, including both macroscopic and microscopic examinations.

The findings from our study and other research, highlight the importance of assessing and monitoring each pathological disorder of the placenta, as it can provide valuable predictive information regarding the occurrence of maternal and neonatal complications. Therefore, a thorough examination of these histopathological changes is not only beneficial but also imperative in predicting and preventing such complications.

Of particular significance, our study revealed that underlying comorbidities in the mother may contribute to the development of pathological lesions in the placenta. In fact, for the first time, we found that a history of malignancy in the mother can be a significant risk factor for placental pathology. Another study demonstrated a correlation between placental calcification and infarction with maternal hypertensive disorders (15).

Contrary to the findings of several studies, our study did not identify a significant association between the history of diabetes mellitus or hypertension and the occurrence of placental pathological disorders. This lack of association may be attributed to the low prevalence of these conditions among the mothers included in our study sample. Our study revealed that placental weight below the 10 ${\;}^{\text{th}}$ percentile (placental hypoplasia) was linked to less common placental disorders, such as perivillous fibrin deposition and delayed villous maturation. We did not identify a statistically significant relationship between placental weight and birth-related disorders, including IUGR, IUFD, and maternal systemic diseases. These findings contradict the findings of several studies, highlighting the need for further research to establish definitive relationships between maternal clinical characteristics and placental pathology.

Our study was subject to certain limitations. First, we lacked access to newborn records, which prevented us from evaluating birth weight and other newborn disorders. Additionally, some of our pathology reports did not include placental weight, further limiting our analysis. Furthermore, this study was conducted on pathology samples from 2 hospitals, which are academic and referral centers for perinatal samples. Therefore, the high prevalence of certain histopathological changes appears reasonable.

## 5. Conclusion

In conclusion, the most prevalent placental pathological changes observed in our study were MVM in 59.6% of cases, followed by the presence of NRBCs in umbilical cord blood vessels during the second half of pregnancy in 16.4% of cases. Placental pathological changes serve as important indicators for maternal and fetal complications, underscoring the significance of early detection in predicting and preventing such complications. Notably, among the maternal medical records, a history of malignancy may serve as an important predictor of placental pathologies. Further academic investigations are warranted to corroborate these findings and elucidate the complex relationship between maternal clinical characteristics and placental pathology.

##  Data Availability

Data supporting the findings of this study are available upon reasonable request from the corresponding author.

##  Author Contributions

S. Sarmadi: Concept and design. E. Mirzaian: Had full access to all of the data in the study and takes responsibility for the integrity of the data and the accuracy of the data analysis, drafting of the manuscript, concept, and design. F. Nili: Acquisition, analysis or interpretation of data, concept, and design. F. Khalafrezaei: Acquisition, analysis or interpretation of data, and drafting of the manuscript. All authors: Critical revision of the manuscript for important intellectual content.

##  Conflict of Interest

The authors declare that there is no conflict of interest.

## References

[bib1] Gude NM, Roberts CT, Kalionis B, King RG (2004). Growth and function of the normal human placenta. Thrombosis Research.

[bib2] Lakshmi Devi CK, Raghupathy NS (2013). The histological findings in human placenta at different gestational ages. IOSR J Dental Med Sci.

[bib3] Redline RW (2008). Placental pathology: A systematic approach with clinical correlations. Placenta.

[bib4] Benton SJ, Lafreniere AJ, Grynspan D, Bainbridge SA (2019). A synoptic framework and future directions for placental pathology reporting. Placenta.

[bib5] Khong TY, Mooney EE, Ariel I, Balmus NC, Boyd TK, Brundler M-A, et al (2016). Sampling and definitions of placental lesions: Amsterdam Placental Workshop Group consensus statement. Arch Pathol Lab Med.

[bib6] Lawn JE, Blencowe H, Pattinson R, Cousens S, Kumar R, Ibiebele I, et al

[bib7] Roberts DJ, Baergen RN, Boyd TK, Carreon CK, Duncan VE, Ernst LM, et al (2023). Criteria for placental examination for obstetrical and neonatal providers. Am J Obstet Gynecol.

[bib8] Redline RW, Roberts DJ, Parast MM, Ernst LM, Morgan TK, Greene MF, et al (2023). Placental pathology is necessary to understand common pregnancy complications and achieve an improved taxonomy of obstetrical disease. Am J Obstet Gynecol.

[bib9] Freedman AA, Keenan-Devlin LS, Borders A, Miller GE, Ernst LM (2021). Formulating a meaningful and comprehensive placental phenotypic classification. Pediatr Dev Pathol.

[bib10] Ernst LM (2018). Maternal vascular malperfusion of the placental bed. APMIS.

[bib11] Heider A (2017). Fetal vascular malperfusion. Arch Pathol Lab Med.

[bib12] Parast MM, Crum CP, Boyd TK (2008). Placental histologic criteria for umbilical blood flow restriction in unexplained stillbirth. Hum Pathol.

[bib13] Suresh SC, Freedman AA, Hirsch E, Ernst LM (2022). A comprehensive analysis of the association between placental pathology and recurrent preterm birth. Am J Obstet Gynecol.

[bib14] Loverro MT, Damiani GR, Di Naro E, Schonauer LM, Laforgia N, Loverro M, et al (2020). Analysis of relation between placental lesions and perinatal outcome according to Amsterdam criteria: A comparative study. Acta Biomed.

[bib15] Siddheshware R, Patil SS, Sambarey PW (2017). Clinical correlation with pathology of placenta in medical disorders of pregnancy and its comparison in normal pregnancy. Int J Reprod Contracept Obstet Gynecol.

[bib16] Saragade P, Chaudhari R, Chakravarti A (2017). Study of histopathological findings of placenta in cases of deliveries at Tertiary Health Care Institute. MVP J Med Sci.

[bib17] Altuncu E, Akman İ, KOTıloğlu E, Başgül A, Yurdakul Z, Demır F, et al (2008). The relationship of placental histology to pregnancy and neonatal characteristics in preterm infants. J Turkish-German Gynecol Assoc.

[bib18] Moscuzza F, Belcari F, Nardini V, Bartoli A, Domenici C, Cuttano A, et al (2011). Correlation between placental histopathology and fetal/neonatal outcome: Chorioamnionitis and funisitis are associated to intraventricular haemorrage and retinopathy of prematurity in preterm newborns. Gynecol Endocrinol.

[bib19] Kumar S, Sudarshan V (2018). The correlation of placental histopathology with neonatal outcome. Tropical J Pathol Microbiol.

[bib20] Azizi M, Rejaei M, Pezeshkpour Z, Alipour MR, Kazemi E, Zare S, et al (2014). Correlation between pathology of placenta and preterm labor: A case-control study. J Biol Todays World.

[bib21] Khong TY, Mooney EE, Nikkels PGJ, Morgan TK, Gordijn SJ (2019). Pathology of the placenta: A practical guide.

[bib22] Langston C, Kaplan C, Macpherson T, Manci E, Peevy K, Clark B, et al (1997). Practice guideline for examination of the placenta: Developed by the placental pathology practice guideline development task force of the college of American pathologists. Arch Pathol Lab Med.

